# Diabetes Mellitus and Its Association with Adverse In-Hospital Outcomes in Patients with COVID-19—A Nationwide Study

**DOI:** 10.3390/v15081627

**Published:** 2023-07-26

**Authors:** Volker H. Schmitt, Lukas Hobohm, Ingo Sagoschen, Visvakanth Sivanathan, Omar Hahad, Christine Espinola-Klein, Thomas Münzel, Karsten Keller

**Affiliations:** 1 Department of Cardiology, University Medical Center of the Johannes Gutenberg-University Mainz, 55131 Mainz, Germany; volker.schmitt@unimedizin-mainz.de (V.H.S.); lukas.hobohm@unimedizin-mainz.de (L.H.); ingo.sagoschen@unimedizin-mainz.de (I.S.); omar.hahad@unimedizin-mainz.de (O.H.); espinola@uni-mainz.de (C.E.-K.); tmuenzel@uni-mainz.de (T.M.); 2 German Center for Cardiovascular Research (DZHK), Partner Site Rhine-Main, 55131 Mainz, Germany; 3 Center for Thrombosis and Hemostasis (CTH), University Medical Center of the Johannes Gutenberg-University Mainz, 55131 Mainz, Germany; 4 Department of Gastroenterology, University Medical Center Mainz of the Johannes Gutenberg-University Mainz, 55131 Mainz, Germany; visvakanth.sivanathan@unimedizin-mainz.de; 5 Medical Clinic VII, Department of Sports Medicine, University Hospital Heidelberg, 69120 Heidelberg, Germany

**Keywords:** COVID-19, diabetes mellitus, cardiovascular risk factors, ventilation, intensive care unit

## Abstract

Background: Diabetes mellitus (DM) represents a relevant risk factor regarding morbidity and mortality worldwide. However, only limited data exist regarding the impact of DM on the clinical outcome of patients with COVID-19 infection. Methods: All hospitalized patients with confirmed COVID-19-infection (ICD-code U07.1) during the year 2020 in Germany were included in the present study. Patients were stratified regarding the co-prevalence of DM (ICD-codes E10-E14), and the impact of DM on in-hospital case fatality and in-hospital adverse events was analyzed. Results: Overall, 176,137 hospitalizations with confirmed COVID-19 infection were documented; of these, 45,232 (25.7%) patients had an additional diagnosis of DM. Diabetic patients with COVID-19 were more often of male sex and 7 years older (median 76.0 (IQR: 66.0–83.0) vs. 69.0 (52.0–81.0) years, *p* < 0.001). COVID-19 patients with DM demonstrated an aggravated comorbidity profile, as reflected by a higher Charlson comorbidity index (6.0 (IQR: 4.0–8.0) vs. 3.0 (1.0–5.0), *p* < 0.001). Risk for pneumonia (OR 1.38 (95% CI: 1.35–1.41), *p* < 0.001), acute respiratory distress syndrome (OR 1.53 (95% CI: 1.47–1.60), *p* < 0.001), and need for intensive care (21.3% vs. 13.3%, *p* < 0.001) were increased in DM patients. DM was an independent risk factor for acute kidney failure (OR 1.49 (95% CI: 1.44–1.53), *p* < 0.001), dialysis (OR 1.56 (95% CI: 1.47–1.66), *p* < 0.001), mechanical ventilation (OR: 1.49 (95% CI: 1.43–1.56), *p* < 0.001), extracorporeal membrane oxygenation (OR 1.44 (95% CI: 1.27–1.62), *p* < 0.001), major adverse cardiac and cerebrovascular events (OR: 1.24 (95% CI: 1.20–1.27), *p* < 0.001), and in-hospital mortality (OR: 1.26 (95% CI: 1.22–1.30), *p* < 0.001). Conclusions: In patients with COVID-19-infection, DM is a relevant risk factor for adverse events, including mortality. The vulnerable patient group of diabetics with COVID-19 requires intense medical care and monitoring during hospitalization.

## 1. Introduction

The first case of infection with severe acute respiratory syndrome coronavirus 2 (SARS-CoV-2) in Germany was documented and reported in January 2020 [1,2,3], approximately two months after the first pneumonia patient cases of unknown origin were reported in China [4,5]. Coronavirus disease 2019 (COVID-19) spread from the first reported cases in Bavaria across Germany [2,3,6,7]. 

Worldwide, millions of deaths have been caused by the ongoing pandemic of COVID-19 [2,7,8], and lockdown measures were implemented by many governments, including that of Germany, aiming to oppose the increasing numbers of infected individuals [2,7]. As a consequence, not only was interpersonal contact reduced with the goal of handling infection, but the utilization of standard medical care regarding chronic diseases like diabetes mellitus (DM) also declined. In this context, several studies revealed decreased hospital admission of patients due to and with chronic cardiovascular diseases and acute cardiovascular events, since health care was focused on COVID-19 management [9,10,11]. The same observation was made regarding cancer [12,13,14], mental/behavioral disorders, and other ailments [14]. The decline in hospital admissions during the pandemic was supposed to be multifactorial, including patients’ fear of COVID-19 disease and the possibility of being infected in hospital [9,15].

Soon after the COVID-19 disease emerged, infected people with DM were shown to be associated with an unfavorable outcome. Diabetic patients suffering from COVID-19 were more likely to need hospital admission and had a higher risk of mortality [16]. However, data on the influence of DM are still fragmentary, vary highly in the literature, and barely exist in many areas worldwide. The present study investigated the impact of DM in patients with COVID-19 on morbidity and mortality based on all hospitalized patients with COVID-19 in Germany during the entire year of 2020.

## 2. Methods

### 2.1. Data Source

Statistical analyses reported in the present study were performed on our behalf by the Research Data Center (RDC) of the Federal Bureau of Statistics (Wiesbaden, Germany). Aggregated and summarized statistics were thereafter provided by the RDC on the basis of our generated SPSS codes (IBM Corp. Released 2011. IBM SPSS Statistics for Windows, Version 20.0. IBM Corp: Armonk, NY, USA), which we sent to the RDC (source: RDC of the Federal Statistical Office and the Statistical Offices of the federal states, DRG Statistics 2020, own calculations) [2,3,17]. 

With the present study analyzing the German nationwide inpatient sample, we aimed to investigate temporal trends of hospitalized patients with a confirmed COVID-19 diagnosis (ICD-code U07.1) during the observational period between 1 January and 31 December 2020, stratifying these included COVID-19 hospitalization cases for co-prevalence of diabetes mellitus (ICD-codes E10-E14). In addition, the impact of diabetes mellitus on treatments and outcomes of COVID-19 patients was calculated. 

### 2.2. Study Oversight, Support, and Ethical Statement

There was no commercial support for the present study and no foreign influence on the preparation of this paper/report. Since our study did not involve direct access by us (as the investigators) to individual patient data and we only had access to aggregated/summarized results provided by the RDC, the approval of an ethics committee and patient informed consent were not required, in accordance with German law [17].

### 2.3. Coding of Diagnoses, Procedures, and Definitions

Shortly after the beginning of the new century in 2004, diagnosis- and procedure-related remuneration was introduced and implemented in the German healthcare system for German hospitals. Coding according to the German Diagnosis-Related Groups (G-DRG) system with the coding of patient data on diagnoses, coexisting conditions, and surgeries/procedures/interventions remains a requirement. Transferring of this coding to the Institute for the Hospital Remuneration System is mandatory for German hospitals to receive remuneration regarding rendered and provided services [3,7]. For this purpose, patient diagnoses are coded according to the International Statistical Classification of Diseases and Related Health Problems (10th revision with German modification, ICD-10-GM), and diagnostic/interventional/surgical procedures are coded according to special OPS codes (Operationen- und Prozedurenschlüssel) [3,7]. With the present study analyzing the German nationwide inpatient sample, we identified all patients with confirmed COVID-19 diagnosis (ICD-code U07.1) hospitalized in German hospitals during 2020 (COVID-19 as the main or secondary diagnosis). 

Obesity is defined as body mass index (BMI) ≥30 kg/m^2^ according to the World Health Organization. Post-COVID status was defined as the status of having previously survived COVID-19 infection before hospitalization with an actual (and therefore recurrent) COVID-19 infection.

### 2.4. Study Outcomes and Adverse In-Hospital Events

The primary study outcome was case fatality, with death of all causes during in-hospital stay (in-hospital case fatality). In addition, we analyzed the prevalence of major adverse cardiovascular and cerebrovascular events (MACCE, composite of all-cause in-hospital death, acute myocardial infarction (ICD code I21), and/or ischemic stroke (ICD code I63)), as well as that of the in-hospital adverse pneumonia events (ICD codes J12–J18), acute respiratory distress syndrome (ARDS, ICD code J80), VTE (ICD codes I26 and I80–I82), acute renal failure (ICD code N17), myocarditis (ICD code I40), myocardial infarction (ICD codes I21–I22), ischemic or hemorrhagic stroke (ICD codes I61–I64), cardiopulmonary resuscitation (CPR, OPS code 8–77), intensive care unit admission (ICU, OPS codes 8–980, 8–98d, and 8–98f), mechanical ventilation (OPS codes 8–71), gastrointestinal bleeding (ICD code K92.0–K92.2), intracerebral bleeding (ICB, ICD code I61), and transfusion of erythrocytes (OPS codes 8–800).

### 2.5. Statistical Analysis

To compare COVID-19 patients with and without DM, we analyzed differences between these two patient groups. Differences in patient characteristics between the groups of hospitalized COVID-19 patients with and without DM were calculated with the Wilcoxon–Whitney U test for continuous variables and Fisher’s exact or chi^2^ test for categorical variables, as appropriate. 

Temporal trends of total numbers of hospitalizations of COVID-19 patients with DM, as well as in-hospital mortality over time and with increasing age, were analyzed and visualized.

Univariate and multivariate logistic regression models were analyzed for the investigation regarding associations between DM and in-hospital adverse events and critical invasive treatments. The multivariate regression models were adjusted for age, sex, cancer, heart failure, coronary artery disease, peripheral artery disease, chronic obstructive pulmonary disease, essential arterial hypertension, obesity, hyperlipidemia, renal insufficiency (GFR < 60 mL/min/1.73 m^2^), and atrial fibrillation/flutter. This epidemiological approach regarding the adjustment was chosen to test the widespread independence of the impact of DM on adverse in-hospital events in light of the possible co-prevalence of these outstanding known predictors of case-fatality rate during hospitalization. The results are presented as odds ratios (ORs) and 95% confidence intervals (CIs). Regarding the logistic regression models, only *p* values < 0.05 (two-sided) were considered to be statistically significant.

All statistical analyses were carried out with the use of SPSS software (IBM Corp. Released 2011. IBM SPSS Statistics for Windows, Version 20.0. IBM Corp: Armonk, NY, USA). 

## 3. Results

### 3.1. Baseline Characteristics

**Figure 1:**
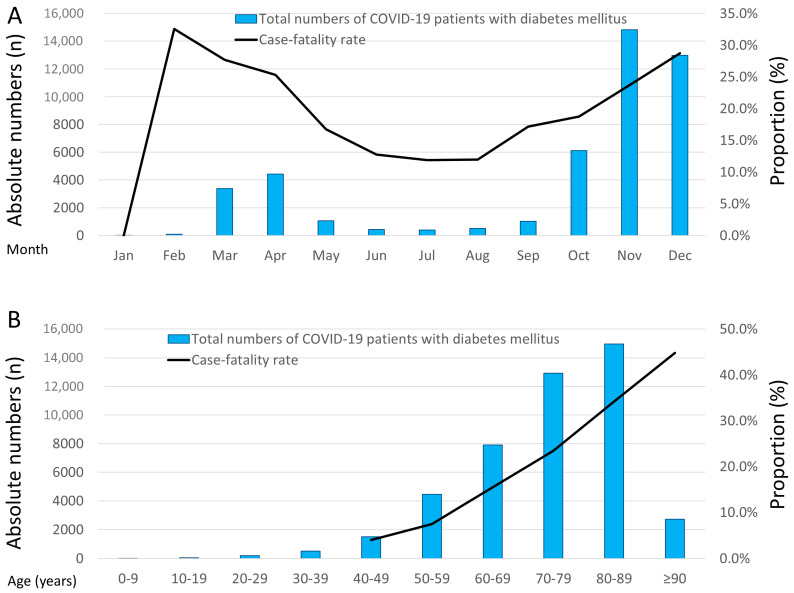
Temporal trends in COVID-19 patients with diabetes mellitus. (**A**) Total numbers of COVID-19 patients with diabetes mellitus and in-hospital case fatality stratified by months of the year 2020. (**B**) Total numbers of COVID-19 patients with diabetes mellitus and in-hospital case-fatality during the year 2020 stratified by age decades.

A total of 176,137 hospitalizations of COVID-19 patients were counted in Germany during the year 2020 and were included in the present analysis. Among these hospitalizations of COVID-19 patients, 45,232 (25.7%) were additionally coded with DM. The highest monthly numbers of hospitalizations of COVID-19 patients were observed in the last quarter of the year 2020. In addition, hospitalizations of COVID-19 patients with DM grew with inclining age (Figure 1).

### 3.2. Comparison Regarding Patient Characteristics of COVID-19 Inpatients with and without Diabetes Mellitus

COVID-19 patients with DM were a median of 7 years older than non-DM patients (76.0 (IQR: 66.0–83.0) vs. 69.0 (52.0–81.0) years, *p* < 0.001) and more often of male sex. 

All investigated cardiovascular risk factors, as well as cardiovascular diseases such as coronary artery disease (24.9% vs. 10.9%, *p* < 0.001) and peripheral artery disease (6.9% vs. 1.9%, *p* < 0.001) were more common in COVID-19 patients with DM than in those without DM (Table 1). In addition, chronic obstructive pulmonary disease, chronic renal insufficiency, and liver diseases were more prevalent in patients with DM. The aggravated comorbidity profile of COVID-19 patients with DM was illustrated by a markedly higher Charlson comorbidity index score in this group (6.0 (IQR: 4.0–8.0) vs. 3.0 (IQR: 1.0–5.0), *p* < 0.001) (Table 1).

### 3.3. Comparison Regarding Respiratory Manifestation of COVID-19 Inpatients with and without Diabetes Mellitus

COVID-19 patients with DM had higher rates of pneumonia (69.1% vs. 57.8%, *p* < 0.001), ARDS (9.3% vs. 5.7%, *p* < 0.001), and multisystemic inflammatory syndrome COVID-19 infection (0.4% vs. 0.2%, *p* < 0.001). Post-COVID-19 status was comparable between the two groups (Table 1).

In the logistic regression analyses, DM was independently associated with both pneumonia (OR: 1.38 (95% CI: 1.35–1.41), *p* < 0.001) and ARDS (OR: 1.53 (95% CI: 1.47–1.60), *p* < 0.001) (Table 2).

### 3.4. Comparison Regarding Treatment Approaches in COVID-19 Inpatients with and without Diabetes Mellitus

The proportion of COVID-19 patients treated in intensive care unit (ICU) admission was 8% higher if DM was present (21.3% vs. 13.3%, *p* < 0.001). While the need for mechanical ventilation was nearly doubled in patients with DM (9.8% vs. 5.9%, *p* < 0.001), the use of extracorporeal membrane oxygenation (ECMO) was only slightly higher in patients with DM in comparison to those without DM (1.0% vs. 0.8%, *p* < 0.001). Acute kidney failure occurred more than twice as often in patients with than in those without DM (19.5% vs. 10.1%, *p* < 0.001), and the need for dialysis was more than doubled in this vulnerable patient group (5.4% vs. 2.4%, *p* < 0.001) (Table 1).

Logistic regression analyses revealed an independent association between DM on the one hand and acute renal failure (OR: 1.49 (95% CI: 1.44–1.53), *p* < 0.001), dialysis (OR: 1.56 (95% CI: 1.47–1.66)), *p* < 0.001), mechanical ventilation (OR: 1.49 (95% CI: 1.43–1.56), *p* < 0.001), and ECMO (OR 1.44 (95% CI: 1.27–1.62], *p* < 0.001) on the other hand (Table 2).

### 3.5. Comparison Regarding Outcomes of COVID-19 Inpatients with and without Diabetes Mellitus

The in-hospital case-fatality rate was 8.6% (24.3% vs. 15.7%, *p* < 0.001), and the MACCE rate was 9.6% (27.0% vs. 17.4%, *p* < 0.001) higher in patients with than in those without DM (Table 1). Multivariate logistic regressions confirmed that DM was independently associated with increased in-hospital case fatality (OR: 1.26 (95% CI: 1.22–1.30), *p* < 0.001) and MACCE (OR: 1.24 (95% CI: 1.20–1.27), *p* < 0.001) (Table 2).

The prevalence of myocardial infarction and stroke was higher in patients with DM (Table 1). However, DM was independently associated with stroke (OR: 1.17 (95% CI: 1.082–1.26), *p* < 0.001) but not with myocardial infarction (OR: 1.06 (95% CI: 0.98–1.15], *p* = 0.176) (Table 2).

While DM did not affect prevalence of venous thromboembolism, all bleeding events were more often detected in COVID-19 patients with DM (Table 1). In the logistic regressions, DM was only independently associated with necessity regarding transfusion of blood constituents (OR: 1.16 (95% CI: 1.11–1.20), *p* < 0.001) but not with intracerebral and gastrointestinal bleeding or with the occurrence of venous thromboembolism (Table 2).

### 3.6. Temporal and Regional Trends

The case-fatality rate of COVID-19 patients with DM was highest during the spring months of February, March, and April, as well as in the winter months of November and December 2020. The case-fatality rate increased with rising age and exceeded 40% in the group of patients aged 90 years and older (Figure 1).

**Figure 2:**
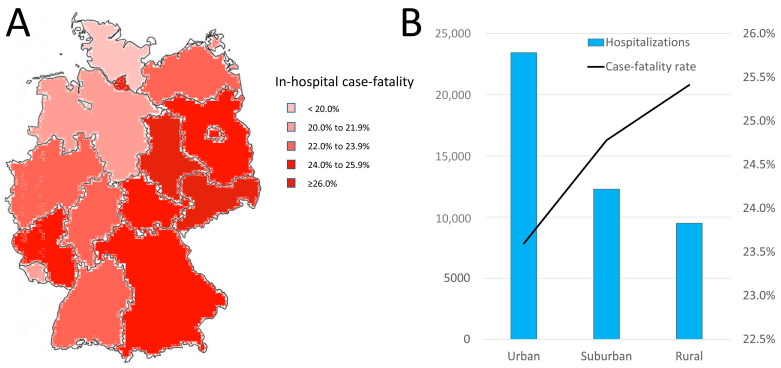
Regional differences regarding case-fatality rate of COVID-19 patients with diabetes mellitus during the year 2020. (**A**) Regional differences regarding the case-fatality rate of COVID-19 patients with diabetes mellitus stratified by federal states of Germany. (**B**) Regional differences regarding total numbers of hospitalizations, as well as the case-fatality rate of COVID-19 patients with diabetes mellitus, stratified by area of Germany.

The analyses revealed regional differences regarding the mortality of COVID-19 patients with DM. The highest in-hospital case-fatality rates were observed in the federal states of Hamburg, Saxony, and Saxony-Anhalt, while the lowest rates were detected in Schleswig-Holstein and Bremen (Figure 2). The most COVID-19 patients were treated in hospitals in urban areas, with the lowest case-fatality rate. In contrast, in hospitals in rural areas, the fewest patients were treated, with high case fatality (Figure 2).

## 4. Discussion

DM is a chronic metabolic disorder characterized by high blood glucose levels. Patients with DM have an increased risk of severe COVID-19 illness and mortality. The underlying mechanisms may involve immune dysfunction, endothelial dysfunction, and proinflammatory cytokine storm, which can exacerbate COVID-19 pathophysiology. In the present study, COVID-19-infected patients with DM were found to have a significantly worse clinical profile, with a higher prevalence of cardiovascular risk factors and comorbidities; a higher risk of a more severe progression of COVID-19 disease, including the need for intensive care therapy, mechanical ventilation, and ECMO; an increased risk of in-hospital adverse events like acute kidney failure and MACCE; and an elevated risk of in-hospital mortality.

In the present study, more than one-quarter of COVID-19 hospitalizations were additionally coded with DM in Germany during the year 2020. Worldwide, the reported prevalence of DM in patients with COVID-19 varied widely [18]. Several studies from China reported a varying prevalence of 7 to 21% [19]. Data from the United States and Spain reported a prevalence of 10% [20,21], whereas in Italy, Mexico, and the United Kingdom, prevalence rates of DM in COVID-19 patients of 17%, 18%, and 20.5% were recorded, respectively [22,23,24]. In a case series comprising 5700 hospitalizations with COVID-19 from New York, USA, a DM prevalence of 33% was reported [25], and in a study from India comprising 401 COVID-19 patients, 47% had additional DM [26]. A meta-analysis by Singh et al. including 14 studies found an estimated pooled prevalence of DM of 11.5% in COVID-19 patients [27]. Li et al. found an overall prevalence of DM of 14.7%, whereas the DM prevalence was 10.4% in non-hospitalized cases and 21.4% in hospitalized COVID-19 cases [28]. The varying prevalence of DM in patients with COVID-19 remains unclear. 

Regarding the data reported in the present study, it must be considered that solely hospitalized cases were investigated, so only the prevalence of DM in hospitalized COVID-19 patients was assessed. This is likely to deviate from the DM prevalence of all COVID-19 patients in Germany, since COVID-19 patients with DM may face a more severe course of disease with a consequent more likely need for hospitalization. The vulnerable group of patients with DM are also often in regular medical attendance and may be admitted to the hospital earlier than patients without such regular medical assistance. However, DM seems not to elevate the risk of COVID-19 infection [18,29].

The findings of the present study of DM as an independent risk factor in patients with COVID-19 infection for a severe course of the disease, morbidity, and mortality are supported by the results of previous studies. An investigation conducted in the United Kingdom revealed DM as an independent risk factor for in-hospital death in patients with COVID-19. In this study, risk for in-hospital death of COVID-19-infected patients was elevated by 2.9-fold in patients with additional type 1 DM and by 1.8-fold in type 2 DM patients [30]. Similar results were reported in a Chinese study in which DM patients with COVID-19 were found to have a 2.95-fold increased risk of death compared to non-diabetics [31]. Data from other countries also show higher mortality in COVID-19 patients in the presence of DM, including Scotland [32], Poland [33], and Saudi Arabia [34]. A meta-analysis comprising seven studies revealed DM as a significant risk factor for suffering from a severe course of COVID-19 infection, with an increased relative risk of 2.11-fold compared to non-diabetic COVID-19 patients. In the same assessment, mortality was also investigated; two studies were included in this analysis, without revealing a significantly elevated risk of mortality due to DM in patients with COVID-19 [27]. In critically ill COVID-19 patients aged ≥70 years who were admitted to an intensive care unit, the presence of DM was associated with higher rates of frailty, relevant comorbidities like chronic heart failure or chronic kidney disease, and an 18% elevated 90-day mortality rate [35]. In their systematic review including 729 studies and more than 29 million COVID-19 patients, Li et al. calculated that in COVID-19-infected patients, 9.5% of severe COVID-19 disease courses and 16.8% of COVID-19-related deaths are attributed to the presence of DM. Furthermore, the group revealed a significant gap between low- and high-income countries, with a more extensive disease burden and, hence, higher attribution to morbidity and mortality of DM in COVID-19 patients in countries with lower income, less health care access, and a lower quality index [28].

Pathophysiologically, DM and SARS-CoV-2 induce similar mechanisms, which may lead to an exacerbation of one or the other. Both induce inflammation and oxidative stress with consecutive damage of the endothelium [36,37,38]. Additional activation of the coagulation system and platelet activation leads to elevated thrombosis. Hence, both diseases fuel organ damage, including lung impairment and respiratory failure due to the activation of similar pathomechanisms, leading to microangiopathy and small vessel thrombosis including the pulmonary vessels. Furthermore, both entities cause further impairment, like mitochondrial dysfunction, microhemorrhage, hypovolemia, and vascular dysfunction. Hence, SARS-CoV-2 exacerbates diabetes-caused immune dysfunction, as well as cardiovascular, renal, and neurological complications [36]. In this context, it was shown that not only the presence of DM but also glycemic control represents a significant factor regarding morbidity and mortality in COVID-19 patients. In a large cohort from England, Holman et al. detected higher mortality rates in COVID-19 patients with high HbA1c values, with a 2.2 and 1.6 elevated risk for mortality with an HbA1c of 10% or more compared to HbA1c of 6.5–7.0% in type 1 and type 2 patients with DM, respectively [39]. Even risk of infection with COVID-19 seems to be linked to glucometabolism. A previous study found a dose-dependent increase in risk of COVID-19 with increasing HbA1c levels. Furthermore, people with undiagnosed DM and known DM with poor glycemic control (HbA1c 8.6% and more) had an elevated risk of COVID-19 [40]. In this context, in a systemic review and meta-analysis, a portion of more than 14% undiagnosed DM was found in hospitalized COVID-19 patients [41]. In a retrospective study including more than 15,000 patients, the intake of metformin was associated with reduced mortality in women with obesity or type 2 DM who were hospitalized due to COVD-19 infection. No reduction in mortality was found in men in this study [42]. An additional study investigated the application of insulin in hyperglycemic COVID-19 patients, and insulin infusion led to a lower risk of severe disease compared to patients without insulin therapy [43]. However, antidiabetic medication needs to be carefully considered in each diabetic patient with regard to disease severity, blood glucose, and other components that may aggravate adverse events [44]. Glycemic control seems to be a relevant factor in the interplay between DM and SARS-CoV-2 infection, reflecting the magnitude of similarity of effected pathways of both diseases. A recent systemic review investigated further risk factors for the poor outcome of COVID-19 patients with DM. Within the group of patients with DM, in their study, Javid et al. identified Black and Asian ethnicity, male sex, high BMI, and older age as parameters corresponding to poorer outcomes of COVID-19 infection [45]. However, patients with DM and COVID-19 represent a vulnerable group who require particular focus from medical care providers. In this context, patients with DM infected by COVID-19 have an elevated risk of ketoacidosis [46,47]. On the other hand, infection with COVID-19 was shown to trigger type 2 DM, and glucose monitoring after COVID-19 infection is discussed in the literature [48,49,50]. The high proportion of 25.7% of hospitalized COVID-19 patients with DM in Germany suggests that DM is associated with more severe disease course [18,19,20,21,22,23,24,27,28].

Thus, SARS-CoV-2 and DM underlie a complex interplay and mutually aggravate often similar pathomechanisms. Hence, the coincidence of the two diseases leads to increased morbidity and mortality, underlining the need for particular focus on this vulnerable patient group.

## 5. Limitations

The present study has some limitations. Due to the nature of an ICD- and OPS-code-based analysis of hospitalized patients, under-reporting and under-coding are possible, and data on concomitant medication or laboratory markers are not available. Furthermore, no follow-up evaluation is available, since data are limited to the time frame of the in-hospital course. In addition, for coding reasons, we were not able to distinguish between new onset of DM and previously existing diagnosis of DM as a comorbidity. 

## 6. Conclusions

In the present study on all hospitalization cases in Germany due to COVID-19 in the year 2020, patients with additional DM were associated with more cardiovascular risk factors and comorbidities; higher risk of a severe course of COVID-19 disease, including intensive care therapy, mechanical ventilation, and ECMO; and an elevated risk of in-hospital adverse events like acute kidney failure and MACCE. In-hospital mortality was also increased in COVID-19 patients with additional DM.

## Figures and Tables

**Table 1:** Characteristics, medical history, presentation, and adverse in-hospital events of the 176,137 hospitalized patients with confirmed COVID-19 infection in Germany in the year 2020 stratified according to diabetes mellitus status.

Parameter	COVID-19 with Diabetes Mellitus (n = 45,232; 25.7%)	COVID-19 without Diabetes Mellitus (n = 130,905; 74.3%)	*p*-Value
Age	76.0 (66.0–83.0)	69.0 (52.0–81.0)	<0.001
Age ≥70 years	30,610 (67.7%)	63,719 (48.7%)	<0.001
Female sex	19,907 (44.0%)	64,042 (48.9%)	<0.001
In-hospital stay (days)	10.0 (5.0–17.0)	7.0 (3.0–13.0)	<0.001
Cardiovascular risk factors			
Obesity	4293 (9.5%)	5090 (3.9%)	<0.001
Essential arterial hypertension	28,752 (63.6%)	53,728 (41.0%)	<0.001
Hyperlipidaemia	12,012 (26.6%)	15,561 (11.9%)	<0.001
Comorbidities			
Coronary artery disease	11,264 (24.9%)	14,310 (10.9%)	<0.001
Heart failure	11,002 (24.3%)	16,117 (12.3%)	<0.001
Peripheral artery disease	3135 (6.9%)	2505 (1.9%)	<0.001
Atrial fibrillation/flutter	12,336 (27.3%)	21,824 (16.7%)	<0.001
Chronic obstructive pulmonary disease	4384 (9.7%)	7770 (5.9%)	<0.001
Chronic renal insufficiency (glomerular filtration rate <60 mL/min/1.73 m^2^)	12,395 (27.4%)	14,977 (11.4%)	<0.001
Cancer	2262 (5.0%)	6739 (5.1%)	0.221
Mild liver disease	655 (1.4%)	990 (0.8%)	<0.001
Severe liver disease	1525 (3.4%)	2614 (2.0%)	<0.001
Charlson comorbidity index	6.0 (4.0–8.0)	3.0 (1.0–5.0)	<0.001
Respiratory manifestations of COVID-19 and post-COVID-19 status			
Pneumonia	31,266 (69.1%)	75,647 (57.8%)	<0.001
Acute respiratory distress syndrome	4190 (9.3%)	7404 (5.7%)	<0.001
Multisystemic inflammatory syndrome COVID-19 infection	172 (0.4%)	325 (0.2%)	<0.001
Post-COVID-19 status	152 (0.3%)	405 (0.3%)	0.384
Treatment			
Intensive care unit	9651 (21.3%)	17,402 (13.3%)	<0.001
Mechanical ventilation	4437 (9.8%)	7705 (5.9%)	<0.001
Extracorporeal membrane oxygenation (ECMO)	449 (1.0%)	1005 (0.8%)	<0.001
Dialysis	2448 (5.4%)	3127 (2.4%)	<0.001
Adverse events during hospitalization			
In-hospital death	10,991 (24.3%)	20,616 (15.7%)	<0.001
Major adverse cardiac and cerebrovascular events (MACCE)	12,210 (27.0%)	22,814 (17.4%)	<0.001
Cardiopulmonary resuscitation	1150 (2.5%)	1709 (1.3%)	<0.001
Venous thromboembolism	1334 (2.9%)	3653 (2.8%)	0.079
Acute kidney failure	8815 (19.5%)	13,260 (10.1%)	<0.001
Myocarditis	54 (0.1%)	172 (0.1%)	0.539
Myocardial infarction	1097 (2.4%)	1656 (1.3%)	<0.001
Stroke (ischaemic or haemorrhagic)	1085 (2.4%)	2111 (1.6%)	<0.001
Intracerebral bleeding	170 (0.4%)	406 (0.3%)	0.035
Gastrointestinal bleeding	904 (2.0%)	2044 (1.6%)	<0.001
Transfusion of blood constituents	4752 (10.5%)	9122 (7.0%)	<0.001

**Table 2:** Impact of diabetes mellitus on in-hospital death and adverse events during in-hospital stay in patients with COVID 19 (univariate and multivariate logistic regression model).

	Univariate Regression Model		Multivariate Regression Model *	
	OR (95% CI)	*p*-Value	OR (95% CI)	*p*-Value
In-hospital death	1.717 (1.673–1.763)	<0.001	1.258 (1.222–1.296)	<0.001
MACCE	1.752 (1.708–1.797)	<0.001	1.236 (1.201–1.271)	<0.001
Pneumonia	1.635 (1.599–1.673)	<0.001	1.380 (1.346–1.414)	<0.001
ARDS	1.703 (1.637–1.772)	<0.001	1.530 (1.466–1.597)	<0.001
Venous thromboembolism	1.059 (0.993–1.128)	0.079	1.021 (0.954–1.092)	0.554
Acute renal failure	2.148 (2.085–2.212)	<0.001	1.486 (1.439–1.534)	<0.001
Myocardial infarction	1.940 (1.796–2.095)	<0.001	1.059 (0.975–1.150)	0.176
Cardiopulmonary resuscitation	1.972 (1.829–2.127)	<0.001	1.476 (1.361–1.601)	<0.001
Stroke (ischemic or hemorrhagic)	1.499 (1.392–1.615)	<0.001	1.170 (1.082–1.264)	<0.001
Intracerebral bleeding	1.213 (1.013–1.451)	0.035	1.096 (0.908–1.322)	0.339
Gastrointestinal bleeding	1.286 (1.188–1.391)	<0.001	0.980 (0.902–1.065)	0.639
Transfusion of blood constituents	1.567 (1.511–1.626)	<0.001	1.157 (1.112–1.204)	<0.001
Mechanical ventilation	1.739 (1.673–1.808)	<0.001	1.494 (1.433–1.558)	<0.001
ECMO	1.296 (1.159–1.449)	<0.001	1.436 (1.270–1.623)	<0.001
Dialysis	2.338 (2.215–2.468)	<0.001	1.559 (1.468–1.655)	<0.001
Post-COVID status	1.086 (0.901–1.310)	0.384	1.029 (0.844–1.256)	0.775

* Adjusted for age, sex, cancer, heart failure, coronary artery disease, peripheral artery disease, chronic obstructive pulmonary disease, essential arterial hypertension, obesity, hyperlipidemia, renal insufficiency (GFR < 60 mL/min/1.73 m^2^), and atrial fibrillation/flutter.

## Data Availability

All code used in this study is publicly available online. The data used in this study are sensitive due to individual patient-level data and will not be made publicly available. The data is available at the Federal Statistical Office of Germany (Statistisches Bundesamt, DEStatis) (source: RDC of the Federal Statistical Office and the Statistical Offices of the federal states, DRG Statistics 2020, and own calculations).
